# Genome-wide characterization, expression analyses, and functional prediction of the NPF family in *Brassica napus*

**DOI:** 10.1186/s12864-020-07274-7

**Published:** 2020-12-07

**Authors:** Jing Wen, Peng-Feng Li, Feng Ran, Peng-Cheng Guo, Jia-Tian Zhu, Jin Yang, Lan-Lan Zhang, Ping Chen, Jia-Na Li, Hai Du

**Affiliations:** 1 College of Agronomy and Biotechnology, Chongqing Engineering Research Center for Rapeseed, Southwest University, Chongqing, 400716 China; 2 Academy of Agricultural Sciences, Southwest University, Chongqing, 400716 China

**Keywords:** *Brassica napus*, *NPF*, Expression analysis, Hormone, Glucosinolate transporter

## Abstract

**Background:**

NITRATE TRANSPORTER 1/PEPTIDE TRANSPORTER (NRT1/PTR) family (NPF) members are essential transporters for many substrates in plants, including nitrate, hormones, peptides, and secondary metabolites. Here, we report the global characterization of NPF in the important oil crop *Brassica napus*, including that for phylogeny, gene/protein structures, duplications, and expression patterns.

**Results:**

A total of 199 *B. napus* (*BnaNPFs*) NPF-coding genes were identified. Phylogenetic analyses categorized these genes into 11 subfamilies, including three new ones. Sequence feature analysis revealed that members of each subfamily contain conserved gene and protein structures. Many hormone−/abiotic stress-responsive *cis*-acting elements and transcription factor binding sites were identified in *BnaNPF* promoter regions. Chromosome distribution analysis indicated that *BnaNPF*s within a subfamily tend to cluster on one chromosome. Syntenic relationship analysis showed that allotetraploid creation by its ancestors (*Brassica rapa* and *Brassica oleracea*) (57.89%) and small-scale duplication events (39.85%) contributed to rapid *BnaNPF* expansion in *B. napus*. A genome-wide spatiotemporal expression survey showed that *NPF* genes of each *Arabidopsis* and *B. napus* subfamily have preferential expression patterns across developmental stages, most of them are expressed in a few organs. RNA-seq analysis showed that many *BnaNPFs* (32.66%) have wide exogenous hormone-inductive profiles, suggesting important hormone-mediated patterns in diverse bioprocesses. Homologs in a clade or branch within a given subfamily have conserved organ/spatiotemporal and hormone-inductive profiles, indicating functional conservation during evolution. qRT-PCR-based comparative expression analysis of the 12 *BnaNPFs* in the NPF2–1 subfamily between high- and low-glucosinolate (GLS) content *B. napus* varieties revealed that homologs of *AtNPF2.9* (*BnaNPF2.12*, *BnaNPF2.13,* and *BnaNPF2.14*), *AtNPF2.10* (*BnaNPF2.19* and *BnaNPF2.20*), and *AtNPF2.11* (*BnaNPF2.26* and *BnaNPF2.28*) might be involved in GLS transport. qRT-PCR further confirmed the hormone-responsive expression profiles of these putative GLS transporter genes.

**Conclusion:**

We identified 199 *B. napus BnaNPFs*; these were divided into 11 subfamilies. Allopolyploidy and small-scale duplication events contributed to the immense expansion of *BnaNPFs* in *B. napus*. The *BnaNPFs* had preferential expression patterns in different tissues/organs and wide hormone-induced expression profiles. Four *BnaNPFs* in the NPF2–1 subfamily may be involved in GLS transport. Our results provide an abundant gene resource for further functional analysis of *BnaNPFs.*

**Supplementary Information:**

The online version contains supplementary material available at 10.1186/s12864-020-07274-7.

## Background

NITRATE TRANSPORTER 1/PEPTIDE TRANSPORTER (NRT1/PTR) homologous proteins are a group of membrane transport proteins present in all major living kingdoms [1–5]. Generally, 12 transmembrane domain (TM) proteins have a conserved structural arrangement connected by short peptide loops, including a large hydrophilic loop between the sixth and seventh TM [5]. In previous studies, homologous proteins were conventionally named according to their first identified substrates, such as NRT (a nitrate transporter), PTR (a peptide transporter), and others [1, 6]. Thereafter, additional substrates of NRT1/PTR homologs were characterized in plants; thus, they were recently and uniformly named as members of the NRT1/PTR family (NPF) [7].

Since AtNPF6.3/AtNRT1.1/CHL1 is characterized as a dual-affinity nitrate transporter in *Arabidopsis* [6, 8, 9], many of its homologs are cloned and functionally characterized in many plant species with multisubstrate transporting capacity. To date, the most well known roles of plant *NPF* genes (*NPFs*) include low- and/or high-affinity nitrate transportation. For example, *Arabidopsis* AtNPF1.1/NRT1.11 and AtNPF1.2/NRT1.12 proteins are low-affinity nitrate transporters involved in redistributing nitrate into developing leaves [10], while *Zea mays* (maize) ZmNPF6.6 is a high-affinity nitrate transporter that can rapidly respond to exogenous nitrate supply [11]. Meanwhile, NPF proteins (NPFs) also behave as nitrite transporters, e.g., *Arabidopsis* AtNPF3.1/Nitr and *Vitis vinifera* VvNPF3.2 [12]. Additionally, NPFs are key transporters for many other substrates, especially hormones and peptides. For example, AtNPF8.1/PTR1 [13, 14], AtNPF8.2/PTR5 [14], and AtNPF8.3/PTR2 [15, 16] are di−/tri-peptide transporters that can mediate the process of flowering, as well as seed and root development; AtNPF4.6/AIT1 transports abscisic acid (ABA) to regulate stomatal aperture [17, 18]; and AtNPF6.3 represses lateral root growth during low nitrate availability by promoting basipetal auxin (IAA) transport [19]. Moreover, members of NPF have been demonstrated to transport secondary metabolites; AtNPF2.10/GTR1 and AtNPF2.11/GTR2 are key transporters for glucosinolate (GLS) [20]. Additionally, a few NPFs display chloride or potassium transport activity: AtNPF2.4 and AtNPF2.5 mediate chloride efflux activity [21, 22], while AtNPF7.3/NRT1.5 regulates pH-dependent K^+^ efflux activity [23].

*Brassica napus* is a significant source of human-edible vegetable oil and animal protein feed; thus, it is an essential oil crop, extensively cultivated in Asia, North America, and Europe. Given essential roles in plant nitrate, di−/tri-peptide, hormone, potassium, chloride, and secondary metabolite transports, NPFs have been systematically identified and analyzed in many species, including *Arabidopsis* [20], *Oryza sativa* (rice) [24], *Triticum aestivum* (wheat) [25], and *Malus domestica* (apple) [26] at the genome-wide level. Identifying and analyzing this gene family in the *B. napus* genome will provide a solid foundation for exploring its potential roles in transporting nitrate, hormones, and GLS, among others.

This study identified NPFs in the *B. napus* genome, accompanied by comprehensive analysis of their gene and protein structural features, chromosomal location, classification, promoter regulation network, and genomic duplication mechanism. Further, we performed systematic expression profile analysis of this gene family in diverse tissues across different developmental stages in *Arabidopsis* (79 tissues) and *B. napus* (50 tissues). Additionally, expression patterns of NPF gene family in *B. napus* under five exogenous hormone inductions (IAA, auxin; ABA, abscisic acid; GA_3_, gibberellic acid; 6-BA, cytokinin; and ACC, ethylene) were assessed, based on the RNA-Seq dataset. Moreover, expression patterns of 12 candidate *NPFs* of the NPF2–1 subfamily in one high- and one low-GLS *B. napus* variety, as well as their expression profiles under hormone induction, were assessed using qRT-PCR. Our study provides an abundant gene resource for further functional analysis of *NPFs* in *B. napus*.

## Results

### Identification and phylogenetic analysis of NPF proteins in *B. napus*

In total, 199 nonredundant NPF protein sequences were obtained in *B. napus* Darmor–*bzh* genome (BnaNPFs) by BLASTP search of the GENOSCOPE dataset (http://www.genoscope.cns.fr/brassicanapus/) [27] and subsequent confirmation by SMART (http://pfam.xfam.org/search/sequence) [28] and PFAM (http://smart.embl-heidelberg.de/smart/show_motifs.pl) [29] analyses (Additional file 1: Table S1). Naming of the candidate BnaNPFs was consistent with previously reported rules [7]. The length of the 199 candidate BnaNPFs ranged from 100 aa (BnaNPF1.4) to 1547 aa (BnaNPF1.9), and the molecular weight ranged from 11.57 kDa (BnaNPF1.4) to 171.42 kDa (BnaNPF1.9). The isoelectric point (pI) ranged from 4.71 (BnaNPF8.13) to 10.23 (BnaNPF2.42), where 40 members had pI values < 7, and 159 members had pI values > 7, suggesting that most of these genes encode alkaline proteins. Subcellular localization prediction by Cell-PLoc2.0 (http://www.csbio.sjtu.edu.cn/bioinf/Cell-PLoc-2/) [30], Pprowler (http://bioinf.scmb.uq.edu.au:8080/pprowler_webapp_1-2/index.jsp) [31], and WoLF PSORT (https://wolfpsort.hgc.jp/) [32] analysis showed that results from these three software tools were highly consistent, and that almost all BnaNPFs are located on the plasmalemma or vacuole (Additional file 1: Table S1). In addition, 100 nonredundant NPF protein sequences were identified in the *Brassica oleracea* genome (BolNPFs) in the BRAD database (http://brassicadb.org/brad/) [33] by the same method (Additional file 2: Table S2). The 53 NPF protein sequences in *Arabidopsis* (AtNPFs) and 94 NPF protein sequences in *Brassica rapa* (BraNPFs) were obtained from a previous study [7] (Additional file 2: Table S2).

**Fig. 1:**
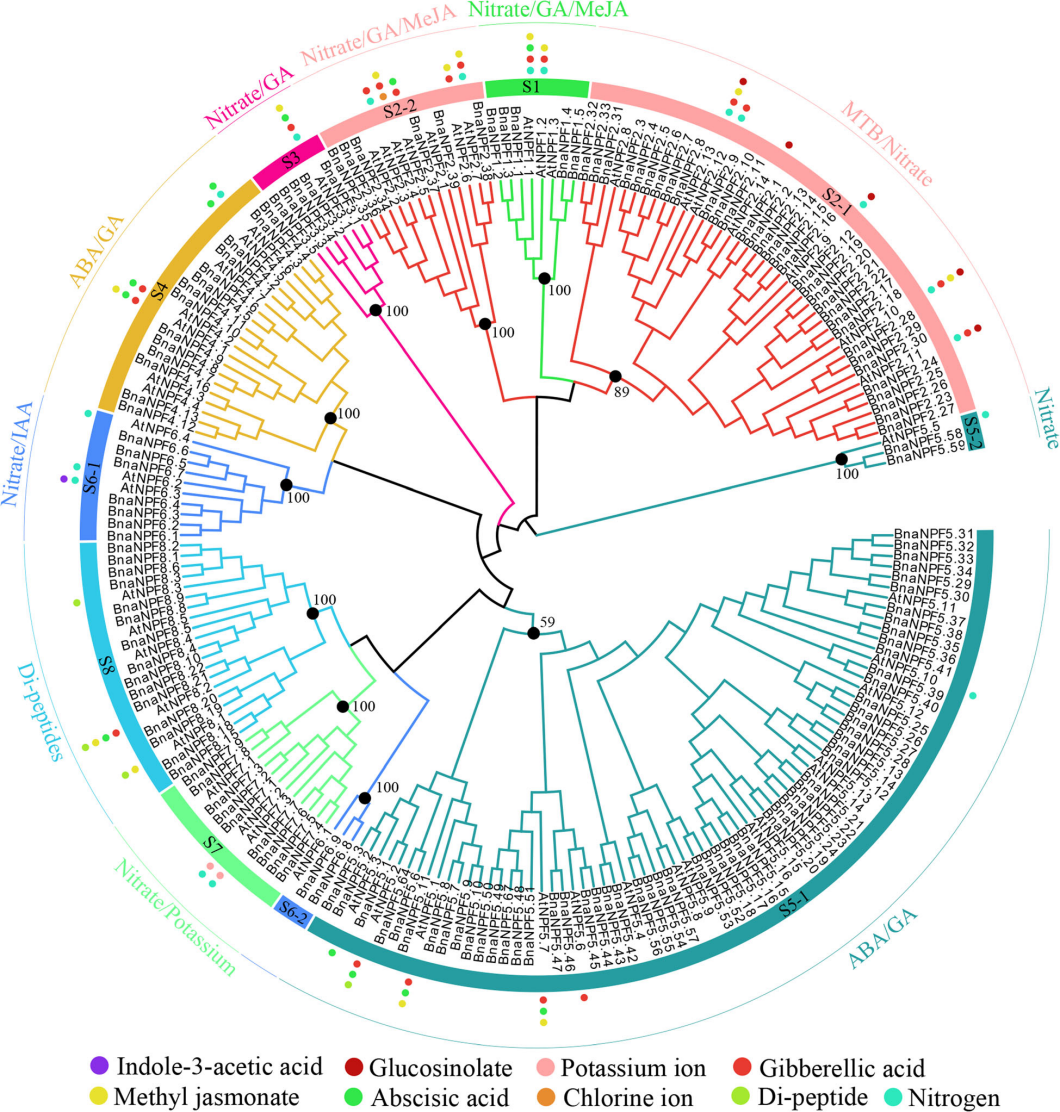
Phylogenetic relationships of *Brassica napus* NPF proteins (BnaNPFs) and *Arabidopsis* NPF proteins (AtNPFs). The neighbor-joining tree (NJ) was built using the full-length NPF proteins in *Arabidopsis* (53) and *B. napus* (155); NPFs were classified into 11 subfamilies (S1, S2–1, S2–2, S3, S4, S5–1, S5–2, S6–1, S6–2, S7, and S8). The bootstrap value of each subfamily is marked in the tree with a black dot. The substrates of AtNPFs that had been functionally demonstrated are indicated in the subfamilies with colored dots, and the main substrates of each subfamily are summarized along the outer circle. IAA: indoleacetic acid, MeJA: methyl jasmonate, ABA: abscisic acid, GA: gibberellic acid, MTB: methylthiobutyl glucosinolate. The neighbor-joining tree was constructed by MEGA7.0 software and visualized and edited in Evolview V3

To explore the classification and evolution of candidate BnaNPFs, multiple sequences of the 53 AtNPFs and the 199 BnaNPFs were aligned using the MAFFT software (https://mafft.cbrc.jp/alignment/server/) [34]. Then, a neighbor-joining (NJ) and maximum-likelihood (ML) phylogenetic tree, were constructed using MEGA 7.0 [35], based on the multiple sequence alignment. Notably, 44 BnaNPFs with large C- or N-terminal deletions were removed from construction of a phylogenetic tree due to lack of common sequence sites; the phylogenetic relationship and classification of these BnaNPFs were predicted by sequence similarity with AtNPFs instead (Additional file 1: Table S1). Based on the topologies and bootstrap support values of the NJ phylogenetic tree, candidate NPFs were divided into 11 subfamilies (Fig. 1). A previous study including 33 plant species divided the NPF family into eight subfamilies (NPF1–NPF8) [7]; three of the eight previously classified subfamilies (NPF2, NPF5, and NPF6 subfamilies) were further divided into two subfamilies in our NJ tree (Fig. 1). Moreover, the results of the NJ and ML trees constructed in this study were highly consistent (Fig. 1 and Additional file 3: Figure S1), demonstrating the reliability of our classification. The distribution of BnaNPFs among different subfamilies was as follows: NPF1 (11), NPF2–1 (36), NPF2–2 (7), NPF3 (7), NPF4 (20), NPF5–1 (71), NPF5–2 (2), NPF6–1 (11), NPF6–2 (2), NPF7 (10), and NPF8 (22). The difference in the number of BnaNPFs in the 11 subfamilies indicated a distinct expansion trend among these subfamilies.

### Protein characteristics and intron pattern diversity

Based on multiple alignment analysis of the 155 full-length BnaNPFs with relatively complete coding regions, the protein sequence feature was further explored. TMs and other conserved protein domains were predicted using the HMMER software (http://www.ebi.ac.uk/Tools/hmmer) [36].

Protein sequence analysis showed that all BnaNPFs contained the PTR2 domain responsible for proton-dependent transport. Moreover, 82.58% (128/155) of BnaNPFs contained 10–12 TMs, 15.48% (24/155) of BnaNPFs had 6–9 TMs, and 0.02% (3/155) of BnaNPFs had 13 TMs. In general, distribution of TMs was conserved in each clade within a subfamily, suggesting functional conservation (Additional file 4: Figure S2). Consistent with a previous report [37], the conserved E_1_X_1_X_2_E_2_R(K) motif was found at the N-terminus of the first TM in 8 of the 11 subfamilies (Additional file 5: Figure S3), though not in S7, S2–2, or S5–2.

At the nucleic acid sequence level, we further analyzed the intron insertion site, number, and phase of candidate *BnaNPFs* by using the Gene Structure Display Server (GSDS) 2.0 (http://gsds.gao-lab.org/) [38]. Our results showed that all 155 *BnaNPFs* contained 1–16 introns, and 86.45% (134/155) of *BnaNPFs* had 3–5 introns (Additional file 6: Figure S4). Notably, three of these introns were highly conserved in almost all *BnaNPFs* in terms of insertion sites and phases; one intron was inserted ahead of the PTR2 domain, and two introns were inserted within the PTR2 domain (one in the third TM and another between the sixth and seventh TMs) (Additional file 6: Figure S4). This finding suggests that these three introns may be necessary for the function of *BnaNPFs*. Moreover, apart from these three introns, the other introns were commonly conserved within each subfamily or clade, but were less conserved among distinct subfamilies (Additional file 6: Figure S4). Furthermore, we found that the intron insertion sites and phases of *BnaNPFs* and *AtNPFs* were highly conserved in each clade or subfamily (Additional file 6: Figure S4), indicating conserved structural features during their evolution.

Overall, the conserved protein and gene sequences strongly support our subfamily division based on phylogenetic analysis.

### Regulatory mechanism in the promoter regions of BnaNPFs

Because c*is*-acting regulatory elements (*CRE*s) in promoter regions are essential for regulating gene transcription levels [39], we predicted the *CRE*s in the promoter regions (− 2000 bp) of *BnaNPFs* using PlantCARE (http://bioinformatics.psb.ugent.be/webtools/plantcare/html/) [40].

**Fig. 2:**
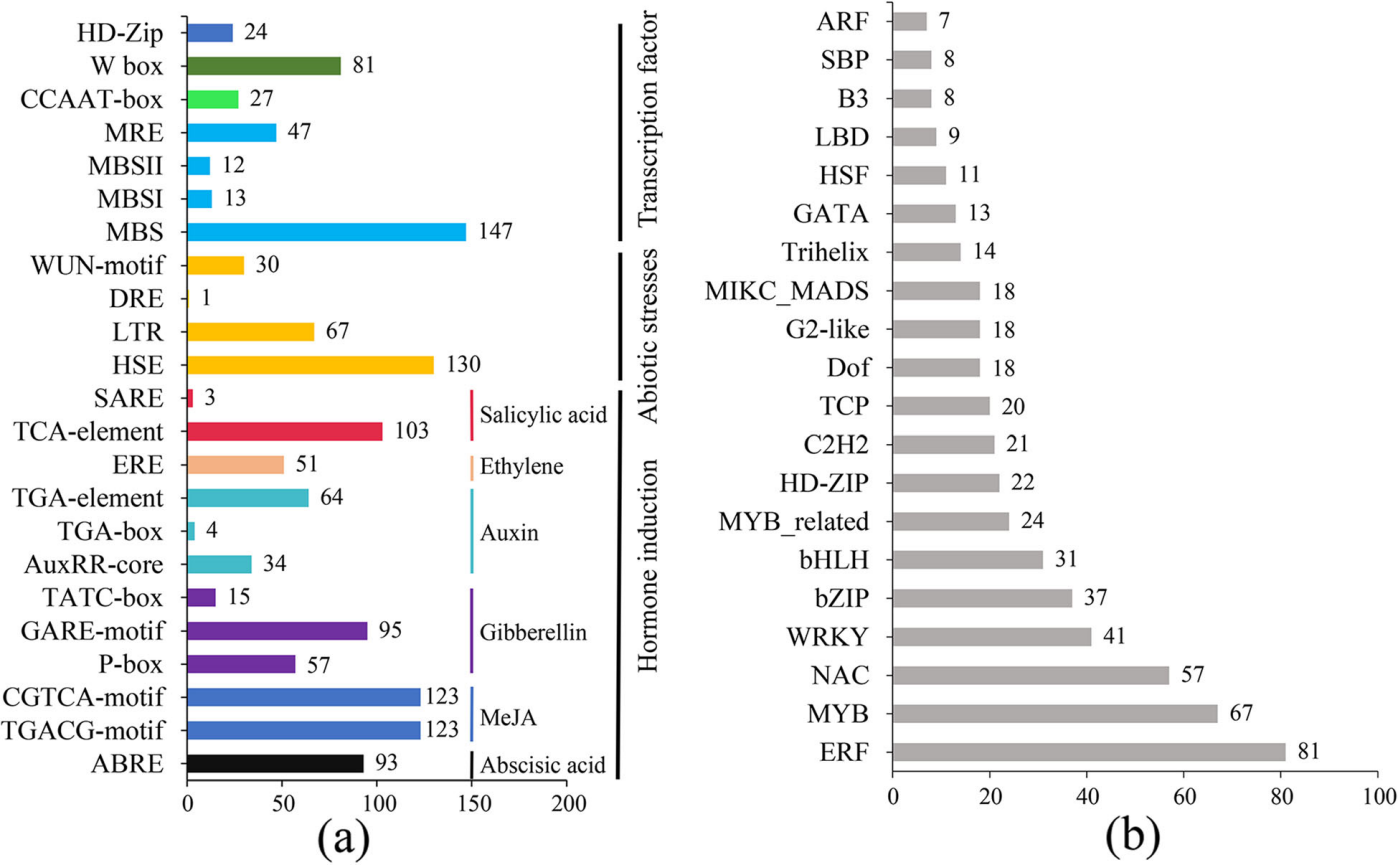
*Cis*-acting regulatory elements (*CRE*s) and transcription factor (TF) binding site analysis in the *BnaNPFs* promoter. **a** The *cis*-elements in the promoter regions of candidate *BnaNPFs*. **b** The top 20 enriched TF gene families that have potential binding sites in the promoter regions of *BnaNPFs*. The abscissa axis of (**a**) and (**b**) represent the numbers of *BnaNPFs* and TFs, respectively. Excel 2016 software was used for data analysis and figure generation

In total, 121 types of *CRE*s were identified in the promoters of the 199 *BnaNPFs*, such as ABA-responsive *cis*-element (ABRE), heat stress-responsive *cis*-element (HSE), and HD-ZIP binding site (HD-Zip) (Fig. 2a; Additional file 7: Table S3). In general, several common *CREs*, such as core elements (CAAT-box and TATA-box) and light-responsive *cis*-element (G-box), were obtained. Meanwhile, a mass of putative *CRE*s that were involved in hormone responses, such as GA, ABA, and ACC, were found in a series of *BnaNPF* promoters (Fig. 2a), suggesting that diverse hormone inductions may regulate their expression. Similarly, many putative *CRE*s associated with abiotic stress, such as HSE (130 of 199 *BnaNPFs*), low temperature-responsive *cis*-element (LTR; 67 of 199 *BnaNPFs*), and wound-responsive *cis*-element (WUN-motif; 30 of 199 *BnaNPFs*), were identified in many *BnaNPF* promoters (Fig. 2a)*.* Furthermore, many transcription factor (TF) binding sites were observed, such as myeloblastosis (MYB) binding sites (MRE, MBS, MBSI, and MBSII) and WRKY binding sites (W box), among others (Fig. 2a).

To further explore the regulatory mechanism of candidate *BnaNPFs*, we inferred the potential regulatory network of *BnaNPFs* using PlantTFDB (http://planttfdb.gao-lab.org/) (Fig. 2b) [41]. Our results showed that up to 582 TFs from 38 TF gene families had potential target binding sites in the promoter regions of *BnaNPFs*. The most enriched TFs belonged to MYB (92 of 582 genes), ethylene response element-binding factor (ERF, 81 genes), NAM-ATAF-CCUC domain-containing protein (NAC, 57 genes), WRKY DNA-binding protein (41 genes), basic leucine zipper (bZIP, 37 genes), and basic helix-loop-helix (bHLH, 31 genes) families (Fig. 2b and Additional file 8: Table S4).

In summary, our results reveal that expression of *BnaNPFs* may be regulated by various kinds of hormones, abiotic stresses, and TFs.

### Chromosomal location and syntenic relationship in BnaNPFs

**Fig. 3:**
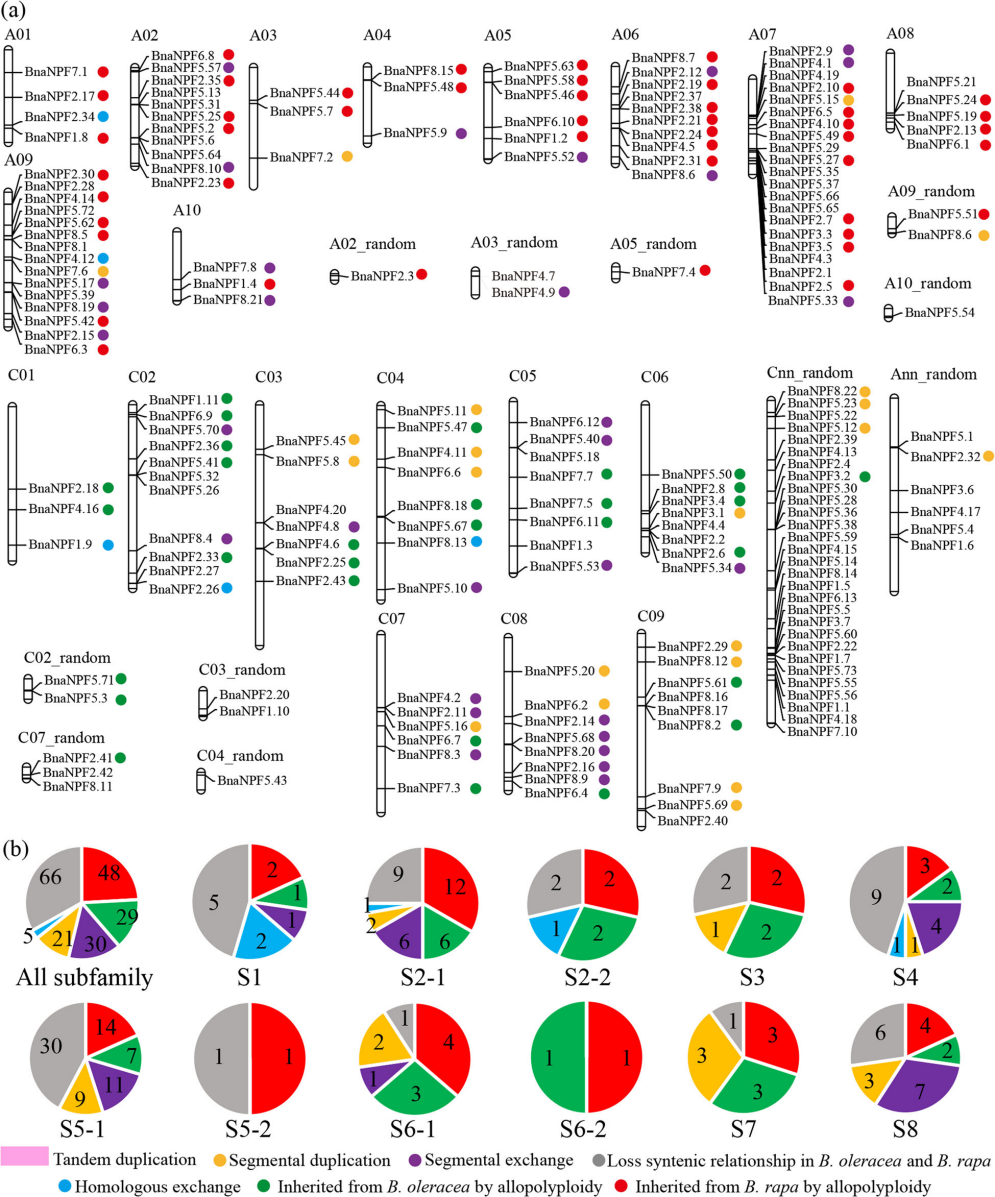
The chromosome location and collinearity relationship of *BnaNPFs*. **a** Chromosome positions of the 199 *BnaNPFs*. The scale of the chromosome is in megabases (Mb). The chromosome number is indicated at the top of each chromosome. **b** The numbers of *BnaNPFs* underwent different duplication events in the 11 subfamilies. The colored dots indicate different duplication events, such as homologous exchange (HE), segmental duplication (SD), etc. The chromosome map of candidate BnaNPFs was drawn by using the MapChart software with default parameters

The distribution of *BnaNPFs* on *B. napus* chromosomes was analyzed based on genomic annotation information obtained from the GENOSCOPE database (http://www.genoscope.cns.fr/brassicanapus/) [27]. As shown in Fig. 3a, most of the 199 *BnaNPFs* were mapped on the 19 chromosomes; however, the exact locations of 6 genes in A_n_ subgenome and 29 genes in the C_n_ subgenome were unclear (Additional file 1: Table S1). The numbers of *BnaNPFs* in A_n_ (95) and C_n_ (104) subgenomes were similar. However, the distribution of *BnaNPFs* on different chromosomes was uneven. For example, A03, A04, and C01 contained only three genes, while A07 had up to 22 genes (Fig. 3a). Notably, *BnaNPFs* belonging to the same subfamily tended to cluster on several chromosomes: 39.44% (28/71) of NPF5–1 subfamily members were distributed on the A02, A07, A09, and C02 chromosomes (Fig. 3a). Similar trends in the NPF gene family were observed in *Arabidopsis*, *B. rapa,* and *B. oleracea*. In *Arabidopsis*, all members of NPF2–2 subfamily (*AtNPF2.1*-*AtNPF2.7*) were clustered on the 03 chromosome, and 46.67% (7/15) of NPF5–1 subfamily (*AtNPF5.10*-*AtNPF5.16*) members were clustered on the 01 chromosome (Additional file 9: Figure S5a); Similarly, members of NPF5–1 subfamily were distributed mainly on the A07 chromosome in *B. rapa* (Additional file 9: Figure S5b) and C07 chromosome in *B. oleracea* (Additional file 9: Figure S5c). These results suggest that *NPF*s in the same subfamily tend to assemble as gene clusters, and this trend may be conserved in plants.

The collinearity of *NPF*s in *B. oleracea*, *B. rapa,* and *B. napus* genomes was analyzed using the CoGe tool (https://genomevolution.org/CoGe/) [42] to explore the expansion mechanism of *BnaNPFs*. Our results show that 133 of 199 *BnaNPFs* in the *B. napus* genome had a syntenic relationship (Fig. 3b; Additional file 10: Table S5). All 133 genes had a collinear relationship with *BraNPFs*, while 127 *BnaNPFs* had a collinear relationship with *BolNPFs*. We further speculated that 49 of the 133 genes (36.84%) were inherited from *B. rapa*, and 28 genes (21.05%) were inherited from the *B. oleracea* genome, based on the syntenic relationship between the descendant and its ancestors. Given that *B. napus* evolved by hybridization between *B. oleracea* and *B. rapa* ~ 7500 years ago, it was evident that allopolyploidy (57.89%) heavily contributed to the rapid expansion of *NPF*s in *B. napus*. Moreover, gene loss following allopolyploidy was biased; the *NPF*s inherited from *B. rapa* were inclined to be retained. Furthermore, 39.85% (56/133) of genes originated from other duplication events within the *B. napus* genome, including 30 genes from segmental exchange (SE), 21 genes from segmental duplication (SD), and 5 genes from homologous exchange (HE) events. These results proved that small-scale duplication events (including HE, SE, and SD) also contributed to the massive expansion of *NPF*s in *B. napus*, especially the SE and SD events. Notably, of the 21 genes that underwent SD events, 15 were derived from *B. rapa*, while the remaining 6 were inherited from *B. oleracea*; this indicates that the genes from *B. rapa* tended to undergo SD in *B. napus*. Regarding the HE event, three of the five HE genes were from the A_n_ subgenome, which replaced the genes in the C_n_ subgenome. This finding confirmed that the A_n_ subgenome replaced more of the C_n_ subgenome after allopolyploidy and featured more dominantly in each chromosome [43]. Three pairs of putative tandem duplication (TD) genes (*BnaNPF2.26*/*BnaNPF2.27*, *BnaNPF4.7*/*BnaNPF4.9*, and *BnaNPF5.22*/*BnaNPF5.23*) were observed, based on their chromosome distribution and sequence similarity.

Overall, our results indicate that allopolyploidy and small-scale duplication events (including SE, SD, and HE) are the primary driving force for the rapid expansion of *BnaNPFs* in *B. napus*, and that those derived from *B. rapa* tended to be retained during evolution.

### Comparative expression analysis of AtNPFs and BnaNPFs across plant development

As gene expression pattern is an essential clue as to its function, in order to explore gene expression patterns as well as expression and function similarity between different species, we analyzed and compared global expression profiles of *AtNPFs* and *BnaNPFs* in different tissues and organs at distinct developmental stages. We used public expression datasets of *Arabidopsis* (http://bar.utoronto.ca/efp/cgi-bin/efpWeb.cgi) [44] and *B. napus* (BioProject ID PRJNA358784).

**Fig. 4:**
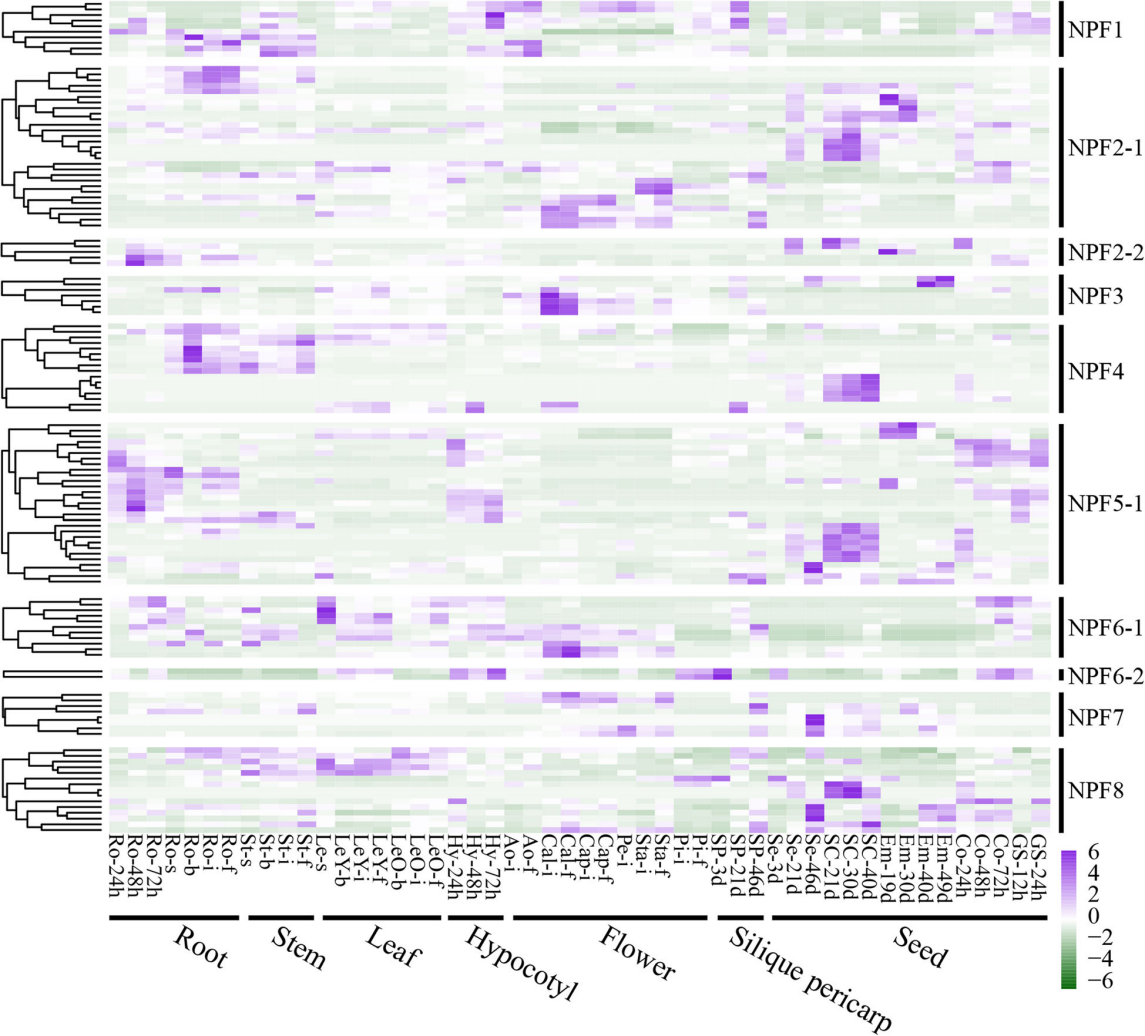
Spatiotemporal expression profiles of *BnaNPFs* across developmental stages by RNA-seq. Ro = root, St = stem, LeY = young leaf, LeO = old leaf, Hy = hypocotyl, Ao = anthocaulus, Cal = calyx, Cap = capillament, Pe = petal, Sta = stamen, Pi = pistil, Sp = silique pericarp, Se = seed, Sc = seed coat, Em = embryo, Co = cotyledon, GS = germination seeds; h, d, s, b, i, and f indicate hour, day, seedling, budding, initial flowering, and full-bloom stages, respectively. Color bar at the bottom represents log2 (FPKM > 1) expression value. The log2 (FPKM > 1) values of BnaNPFs were visualized by the R package and listed in Additional file 11: Table S6

In *B. napus*, with the exception of 67 *BnaNPFs* having no detectable expression values (FPKM < 1) that were excluded from analysis (Additional file 11: Table S6), most (132/199) of the remaining genes had preferential expression profiles in the 50 tissues of seven organs (root, stem, leaf, hypocotyl, flower, silique pericarp, and seed) at six developmental stages (Fig. 4 and Additional file 11: Table S6). For instance, members of the NPF1 subfamily had higher transcriptional levels in root, stem, hypocotyl, flower, and silique pericarp; members of NPF7 were highly expressed in flowers, silique pericarp, and seeds; and members of NPF2–1 were mainly expressed in flower and seed tissues (Fig. 4). In general, expression patterns were conserved in each subfamily or each clade within a subfamily, but were quite different across different subfamilies, suggesting the expression differentiation trend of this gene family. For example, expression patterns of NPF1, NPF2–2, NPF3, and NPF6–1 subfamilies were similar in each subfamily, while the expression profile of the NPF2–1 subfamily was classified into three conserved patterns that were consistent with the three major clades in this subfamily. Additionally, we found that 40% (6/15) of the *BnaNPF*s expressed explicitly in seeds belong to the NPF2–1 subfamily, and 33.33% (5/15) belong to the NPF4 subfamily, suggesting essential roles for these two subfamilies in seed development.

In *Arabidopsis*, consistent with the situation in *B. napus*, most of the *AtNPFs* had preferential expression patterns in the organs investigated (Additional file 12: Figure S6). Members of the NPF2–2 subfamily (*AtNPF2.3*, *AtNPF2.4*, *AtNPF2.5,* and *AtNPF2.7*) were preferentially expressed in roots; *AtNPF4.1* and *AtNPF4.5* in the NPF4 subfamily were mainly expressed in seeds; and *AtNPF2.10* and *AtNPF2.11* in the NPF2–1 subfamily had higher expression levels in roots, stems, leaves, and flowers. Notably, the expression patterns of homologs in both species were generally conserved. Members of NPF2–1 in *B. napus* and *Arabidopsis* were preferentially expressed in flower and seed organs, and members of NPF2–2 in these two species were preferentially expressed in roots. Given that genes with similar expression patterns may share similar functions, the homologs may have similar/conserved functions in *Arabidopsis* and *B. napus*.

### Expression profiles of BnaNPFs under different hormone inductions

As mentioned (Fig. 2), many hormone-responsive *CRE*s were observed in the promoter regions of candidate *BnaNPFs*, suggesting possible roles for plant hormones in *BnaNPF* expression. Therefore, we analyzed the expression profiles of *BnaNPFs* under five exogenous hormone treatments (IAA, ABA, 6-BA, GA_3_, and ACC) in *B. napus* seedling roots, based on the RNA-seq data (BioProject ID: PRJNA608211).

**Fig. 5:**
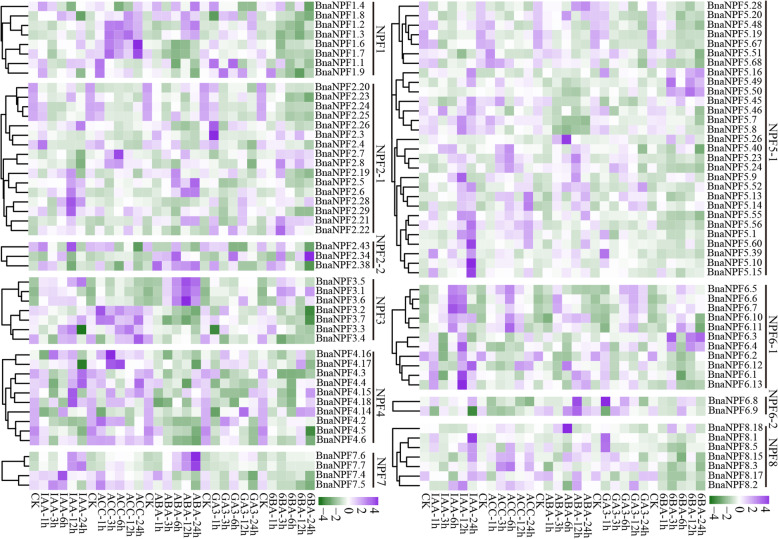
Expression patterns of *BnaNPFs* under five exogenous hormone treatments in *B. napus* roots at seedling-stage. CK represents no hormone treatment (0 h). ACC: 1-aminocyclopropanecarboxylic acid, 6-BA: 6-benzylaminopurine; 1, 3, 6, 12, and 24 represent the number of hours after the treatments. The *BnaNPFs* with no or weak expression levels (FPKM < 1) are not shown. Color bar at the top represents log2 (FPKM > 1) expression values. The log2 (FPKM > 1) values of BnaNPFs were visualized by the R package

Results showed that 32.66% (65/199) of the *BnaNPFs* were upregulated by one or more types of hormones (Fig. 5). With the exception of the NPF5–2 subfamily, which had no detectable expression level, the expression patterns of the other 10 subfamilies were induced at different levels under hormone treatments. NPF1 subfamily members were positively induced by ACC induction; 42.86% (3/7) of NPF3 subfamily members were upregulated by ABA and ACC treatments; and 90.91% (10/11) of NPF6–1 subfamily members had higher expression levels under the five hormone treatments. In contrast, 35.18% (70/199) of *BnaNPFs* were downregulated by these five hormone treatments (Fig. 5), e.g., members of the NPF4 (*BnaNPF4.2*, *BnaNPF4.3*, *BnaNPF4.4*, *BnaNPF4.5*, *BnaNPF4.6*, *BnaNPF4.14*, *BnaNPF4.15*, *BnaNPF4.16*, *BnaNPF4.17,* and *BnaNPF4.18*) and NPF5–1 (*BnaNPF5.19*, *BnaNPF5.20*, *BnaNPF5.28*, *BnaNPF5.48,* and *BnaNPF5.67*) subfamilies. In general, genes in a clade or branch within a given subfamily had similar hormone-induced expression profiles, such as in the NPF2–1 (*BnaNPF2.20, BnaNPF2.23*, *BnaNPF2.24,* and *BnaNPF2.25*) and NPF6–1 (*BnaNPF6.5*, *BnaNPF6.6,* and *BnaNPF6.7*) subfamilies (Fig. 5), implying their functional conservation.

Overall, the expression of many *BnaNPFs* was sensitive to exogenous hormone induction, suggesting that the essential roles of this gene family in diverse plant processes may be regulated by hormone-mediated patterns.

### Expression of BnaNPFs in high- and low-GLS content *B. napus* varieties

**Fig. 6:**
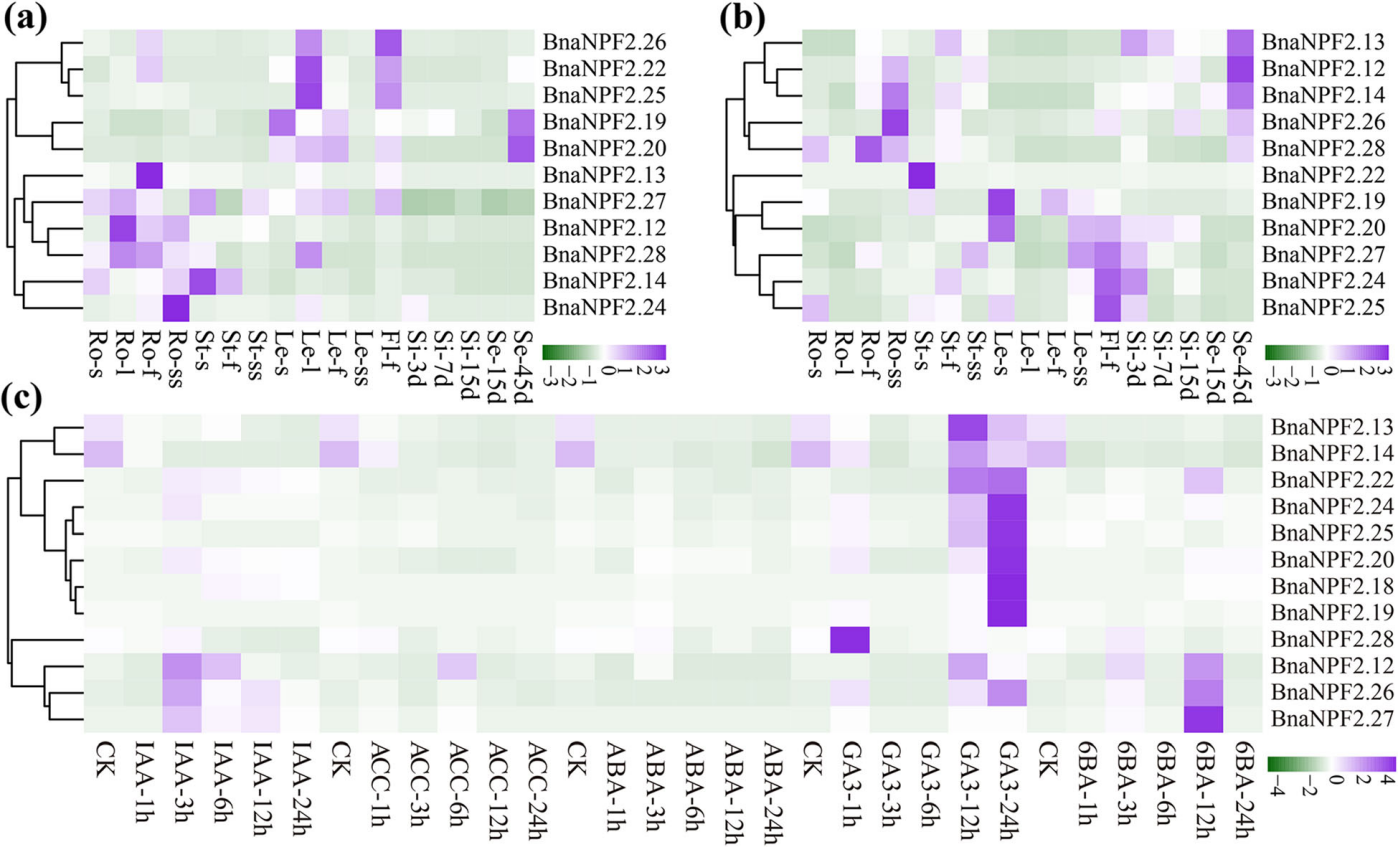
Comparative expression profile analysis of 12 *BnaNPFs* between high- and low-glucosinolate (GLS) content *B. napus* varieties and under hormone treatments. **a–b** Expression profiles of 12 *BnaNPFs* in Zhongshuang 11 (ZS11; low-GLS content) and Zhongyou 821 (ZY821; high-GLS content) by qRT-PCR. Ro = root, St = stem, Le = leaf, Si = silique, Se = seed, Fl = flower; d, s, l, f, and ss indicate day, small seedling at five-leaf stage, large seedling at eight-leaf stage, full-bloom stage, and silique stage, respectively. **c** Expression profiles of 12 *BnaNPFs* under IAA, ACC, ABA, GA_3_, and 6-BA inductions in *B. napus* seedling roots. IAA: auxin; CK represents no hormone treatment (0 h). Color bar at the right represents log2 expression values. The log2 (expression value) of 12 BnaNPFs were visualized by the R package

GLS is a class of important secondary metabolites found in Brassicaceae that have distinctive benefits for plant defense and human nutrition (such as inhibiting carcinogen activation) [45, 46]. Recently, several *Arabidopsis* NPFs, including AtNPF2.10, AtNPF2.11, and AtNPF2.9, were shown to be involved in GLS transport [20, 47]. Phylogenetic analysis showed that these proteins and their *B. napus* homologs (20 proteins, BnaNPF2.12–BnaNPF2.30 and BnaNPF2.40) were clustered into the NPF2–1 subfamily with conserved sequence features (Fig. 1) and expression profiles (Fig. 4), implying that the *B. napus* homologs may have similar roles in GLS transport. To confirm the possible roles of these BnaNPFs in GLS transport, we further compared the expression profiles of 12 GLS-coding genes between low- and high-GLS content *B. napus* varieties, Zhongshuang 11 (ZS11; Fig. 6a and Additional file 13: Table S7) and Zhongyou 821 (ZY821; Fig. 6b and Additional file 13: Table S7), using qRT-PCR [48]. Eight genes in this subfamily that have no detectable expression by RNA-Seq analysis may be pseudogenes, and thus were excluded from this analysis.

Consistent with RNA-seq results (Fig. 4), all 12 candidates were preferentially expressed in a few organs at different developmental stages; however, one gene (*BnaNPF2.18*) was not expressed in any of the samples investigated. Moreover, most of the other 11 candidates exhibited different expression patterns in the root, stem, leaf, flower, silique, and seed organs between the two varieties (Fig. 6a,b). For example, the three homologs of the *AtNPF2.9* gene (*BnaNPF2.12*, *BnaNPF2.13,* and *BnaNPF2.14*) were highly expressed in seed tissues of ZY821 but had no detectable expression levels in the seed tissues of ZS11. Similarly, although the expression levels were relatively lower, two homologs of *AtNPF2.11* (*BnaNPF2.26* and *BnaNPF2.28*) were expressed in seed tissues of ZY821 but were not expressed in those of ZS11. In contrast, the homologs of *AtNPF2.10* (*BnaNPF2.19* and *BnaNPF2.20*) were highly expressed in seed tissues of ZS11 but not in those of ZY821. It was previously reported that *AtNPF2.9* is the typical indole-specific GLS transporter gene, whereas *AtNPF2.11* is the transporter gene for both indole and aliphatic GLS [20, 37]. Given the fact that GLS content in ZS11 seeds is significantly lower than in ZY821, our results imply that these five genes are involved in GLS transport in *B. napus* seed tissues, especially the three more highly expressed in ZY821 seed tissues.

We further confirmed expression patterns of the 12 candidates under five hormone treatments (IAA, ABA, 6-BA, ACC, and GA_3_) in ZS11 via qRT-PCR (Fig. 6c and Additional file 14: Table S8). Similar to the results from RNA-seq analysis (Fig. 5), the expression pattern of these candidates was divided into two major groups: the first group of genes (*BnaNPF2.13*, *BnaNPF2.14*, *BnaNPF2.18*, *BnaNPF2.19*, *BnaNPF2.20*, *BnaNPF2.22*, *BnaNPF2.24*, and *BnaNPF2.25*) was upregulated mainly by GA_3_ and IAA inductions, while the second group of genes (*BnaNPF2.12*, *BnaNPF2.26*, *BnaNPF2.27*, and *BnaNPF2.28*) was upregulated mainly by IAA, GA_3_, and 6-BA treatments. Notably, most members in the first group are homologs of *AtNPF2.9* and *AtNPF2.10*, whereas most members in the second group are homologs of *AtNPF2.11*, suggesting different hormone response patterns.

Overall, these results indicate that the homologs of *AtNPF2.9*, *AtNPF2.10*, and *AtNPF2.11* may be the transporter genes for GLS in *B. napus* seeds, and their function may be impacted by hormone treatments.

## Discussion

To date, a wide range of NPF family substrates have been characterized in plants, including nitrate/nitrite [6], di/tri-peptides [13, 14], hormones (such as IAA, ABA, GA, MeJA, etc.) [18], chloride [21, 22], potassium [23], and secondary metabolites [20, 49], demonstrating their diverse roles in plants. Previously, the plant NPFs were well known for their important roles in nitrate/nitrite transportation (Table 1). Accordingly, up to 10 of the 11 subfamilies of this gene family (NPF1, NPF2–1, NPF2–2, NPF3, NPF4, NPF5–1, NPF5–2, NPF6–1, NPF7, and NPF8) had been shown to be involved in nitrate transport in plants. Moreover, the majority of functionally characterized NPFs in these 10 subfamilies were low-affinity nitrate transporters, while a few members of the NPF1 and NPF6–1 subfamilies acted as high-affinity nitrate transporters, such as AtNPF6.3 and its homolog in *Medicago truncatula* (MtNPF6.8) [6, 11, 13, 69–71]. NPFs were identified as important transporters for hormones as well, with eight subfamilies (NPF1, NPF2–1, NPF2–2, NPF3, NPF4, NPF5–1, NPF6–1, and NPF8) associated with hormone transportation (Fig. 1 and Table 1). Among them, members of the NPF1, NPF2–2, NPF3, NPF4, and NPF5–1 subfamilies have a relatively wider range of substrates and are commonly involved in GA, ABA, and MeJA transports; for example, AtNPF4.1/AIT3 in the NPF4 subfamily can transport ABA, GA_1/3/4/8/20_, and MeJA [17, 18, 50, 55]. Members of the NPF2–1 subfamily, such as AtNPF2.10, were reported to transport two substrates (GA and MeJA) [18, 54]. On the contrary, members of each of the NPF6–1 and NPF8 subfamilies transport only one kind of hormone: AtNPF6.3 (NRT1.1/CHL1) of the NPF6–1 subfamily is involved in IAA transport [19], while AtNPF8.1 of the NPF8 subfamily is related to MeJA transport [18]. Additionally, NPFs can transport many other substrates, including di−/tri-peptides, chloride, potassium, and secondary metabolites. For instance, AtNPF5.2/PTR3 of the NPF5–1 subfamily, and AtNPF8.1, AtNPF8.2, and AtNPF8.3 of the NPF8 subfamily showed specific dipeptide transport activity [13–16, 60]. In terms of chloride transport, AtNPF2.4 and AtNPF2.5 of the NPF2–2 subfamily mediate chloride efflux activity [21, 22]. Regarding potassium transport, AtNPF7.3 displayed pH-dependent K^+^ efflux activity and mediated a proton-coupled H^+^/K^+^ antiporter activity for K^+^ loading into the xylem [23]. Recently, these gene family members were shown to be involved in secondary metabolite transport [20, 49]. For instance, five NPF2–1 subfamily members (AtNPF2.10, AtNPF2.11, AtNPF2.9, AtNPF2.14, and AtNPF2.13/NRT1.7) were shown to be the key transporters for GLS [20, 37]. Similarly, an NPF from the same subfamily in *Catbarantbus roseus*, CrNPF2.9, can transport monoterpene indole alkaloids [49]. Overall, NPFs have diverse substrates in plants, especially for nitrate and multiple hormone transportation.

**Table 1:** Summary of the substrates of plant NPF proteins

Species	Name	Other Name	Gene ID	Subfamily	Substrates	Regulation by hormone
*Arabidopsis*	AtNPF1.1	NRT1.12	At3g16180	NPF1	NO_3_ −[10]; ABA/GA_1/3/4_/MeJA [18]	**–**
	AtNPF1.2	NRT1.11	At1g52190	NPF1	NO_3_^−^ [10]; GA_1/3/4_/MeJA [18, 50]	**–**
	AtNPF2.3	NAXT2	At3g45700	NPF2–2	GA_1/3/4_ [18]; NO_3_ −[51]	**–**
	AtNPF2.4		At3g45690	NPF2–2	Chloride [21]; GA_1/3/4_/MeJA [18]	ABA
	AtNPF2.5		At3g45680	NPF2–2	ABA/GA_1/3/4_ [18]; chloride [22]	**–**
	AtNPF2.6		At3g45660	NPF2–2	GA_1/4_/MeJA [18]	**–**
	AtNPF2.7	NAXT1	At3g45650	NPF2–2	NO_3_ −[52]; GA_1/3/4_/MeJA [18]	**–**
	AtNPF2.9	NRT1.9/GTR3	At1g18880	NPF2–1	NO_3_ −[53]; 4MTB [20]	**–**
	AtNPF2.10	GTR1	At3g47960	NPF2–1	NO_3_^−^/4MTB [20]; 8MTO [47]; GA_1/3/4_/MeJA [18, 54]	MeJA
	AtNPF2.11	NRT1.10/GTR2	At5g62680	NPF2–1	NO_3_^−^/4MTB [20]; 8MTO [47]; GA_3_ [55]	**–**
	AtNPF2.12	NRT1.6	At1g27080	NPF2–1	NO_3_ −[56]; GA_1/3_ [18]	**–**
	AtNPF2.13	NRT1.7	At1g69870	NPF2–1	NO_3_ −[57]; 4MTB [20]; GA_1/3/4_/MeJA [18]	**–**
	AtNPF2.14		At1g69860	NPF2–1	4MTB [20]	**–**
	AtNPF3.1	Nitr	At1g68570	NPF3	NO_3_^−^/NO_2_ −[12]; ABA/GA_1/3/4/8/20_/MeJA [18, 50, 55]	ABA; GA
	AtNPF4.1	AIT3	At3g25260	NPF4	ABA [39]; GA_1/3/4_/MeJA [18, 54]; GA_3/4/8/20_ [55]	**–**
	AtNPF4.2	AIT4	At3g25280	NPF4	GA_1/3_ [18]; ABA [17, 58]	**–**
	AtNPF4.5	AIT2	At1g27040	NPF4	ABA [17, 18, 58]	**–**
	AtNPF4.6	NRT1.2/AIT1	At1g69850	NPF4	NO_3_ −[59]; ABA [17, 18, 58]	**–**
	AtNPF5.1		At2g40460	NPF5–1	ABA/GA_1/3/4_/MeJA [18]	**–**
	AtNPF5.2	PTR3	At5g46050	NPF5–1	ABA/GA_1/3/4_ [9]; di-peptides [60]	SA; MeJA; ABA
	AtNPF5.3		At5g46040	NPF5–1	ABA [18]	**–**
	AtNPF5.5		At2g38100	NPF5–2	NO_3_ −[61]	**–**
	AtNPF5.6		At2g37900	NPF5–1	GA_1/4_ [18]	**–**
	AtNPF5.7		At3g53960	NPF5–1	ABA/GA_1/3/4_/MeJA [18]	**–**
	AtNPF5.10		At1g22540	NPF5–1	NO_3_ −[61]	**–**
	AtNPF6.2	NRT1.4	At2g26690	NPF6–1	NO_3_ −[62]	**–**
	AtNPF6.3	NRT1.1/CHL1	At1g12110	NPF6–1	NO_3_ −[6]; IAA [19]	IAA
	AtNPF7.2	NRT1.8	At4g21680	NPF7	NO_3_ −[53]	**–**
	AtNPF7.3	NRT1.5	At1g32450	NPF7	NO_3_ −[53]; K^+^ [23]	**–**
	AtNPF8.1	PTR1	At3g54140	NPF8	di-peptides [13, 14]; MeJA [18]	**–**
	AtNPF8.2	PTR5	At5g01180	NPF8	di-peptides [14]	**–**
	AtNPF8.3	PTR2/NTR1	At2g02040	NPF8	di-peptides [15, 16]; histidine [63]	**–**
*O.sativa*	OsNPF2.2	OsPTR2	Os12g44100	NPF2–1	NO_3_ −[64]	**–**
	OsNPF2.4		Os03g48180	NPF2–1	NO_3_ −[65]	**–**
	OsNPF7.2		Os02g47090	NPF7	NO_3_ −[66]	**–**
	OsNPF8.9	OsNRT1	Os03g13274	NPF8	NO_3_ −[67]	**–**
*Z. mays*	ZmNPF6.4		GRMZM2G086496_P01	NPF6–1	chloride/ NO_3_ −[11]	**–**
	ZmNPF6.6		GRMZM2G161459_P02	NPF6–1	NO_3_ −[11]	**–**
*C. roseus*	CrNPF2.9		KX372303	NPF2–1	Alkaloid [49]	**–**
*V.vinifera*	VvNPF3.2		GSVIVT01025795001	NPF3	NO_3_^−^/NO_2_ −[12]	**–**
*C. sativus*	CsNPF3.2	CsNitr1	Cucsa.337560.1	NPF3	NO_2_ −[68]	**–**
*M. truncatula*	MtNPF1.7	NIP/LATD	Medtr1g009200	NPF1	NO_3_ −[69]	**–**
	MtNPF6.8	MtNRT1.3	Medtr5g085850	NPF6–1	NO_3_ −[70]	**–**

Notably, NPFs in most subfamilies can generally transport more than one type of substrate (Table 1). For example, NPF2–1 subfamily members can transport four types of substrates: nitrate, GLS, GA, and MeJA (Fig. 1 and Table 1); in *Arabidopsis*, AtNPF6.3 of the NPF6–1 subfamily transports nitrate as well as auxin [19]. Moreover, many studies have found that the role of NPFs in transporting diverse substrates generally demonstrates hormone-mediated characteristics. For example, *AtNPF6.3* of the NPF6–1 subfamily was highly induced by IAA treatment under low nitrogen conditions [72]. *AtNPF2.4* was repressed by ABA treatment and then played a role in chlorine transport [21]. *AtNPF2.10* was upregulated by MeJA treatment, which then accelerated the transport of gibberellin [54]. *AtNPF3.1* was upregulated by ABA to promote the transport of gibberellin [55]. AtNPF5.2 was regulated by SA, MeJA, and ABA treatments against biotic and abiotic stresses [60]. In this study, many *CREs* involved in hormone responses, such as SA- (103/199 genes), ABA- (93/199 genes), and MeJA-responsive *CRE* (123/199 genes), were found in a series of *BnaNPF* promoters (Fig. 2), suggesting their potential hormone-inducing characteristics. Accordingly, the expression of 32.66% of the *BnaNPFs* (65/199 genes) were regulated by one or more types of hormone inductions (ABA, IAA, 6-BA, GA_3_, and ACC) (Fig. 5). Consistent with previous work [72], NPF6–1 subfamily proteins in *B. napus* (e.g., *BnaNPF6.5*, *BnaNPF6.7*, and *BnaNPF6.7*) were also highly induced by IAA in our study. Additionally, we revealed that the genes involved in GLS transport are induced by IAA, GA_3_, and 6-BA treatments (Fig. 6). Together, these results support the hypothesis that hormones have an essential role in substrate transport by NPFs.

Given that the role of NPFs in transporting many substrates is crucial for plant development and stress response, genome-wide analyses of the *NPF* gene family have been performed in many plant species. However, the classification of this gene family is not yet uniform. For example, this gene family was divided into 10 supergroups and 32 groups (subfamilies) based on phylogenetic analysis of 20 plant genomes [73]. In contrast, other research divided this gene family into 8 subfamilies (NPF1-NPF8) based on similar analysis in 33 plant genomes (including *Physcomitrella patens* and *Selaginella moellendorffii*) [7]. Subsequent studies generally followed the criteria of the latter division [25, 26, 74]. Recently, in apple, the NPF2 subfamily was further divided into two groups (subfamilies) [26], implying a new classification trend. In this study, we found that three of the eight previously demonstrated subfamilies (NPF2, NPF5, and NPF6) [7] should be divided into two subfamilies with high bootstrap values: NPF2–1/NPF2–2, NPF5–1/NPF5–2, and NPF6–1/NPF6–2, respectively (Fig. 1). To confirm this result, we further expanded our dataset to include NPFs from *O. sativa*, *Populus trichocarpa*, *Z. mays*, *B. rapa*, *B. oleracea,* and *Glycine max* (Additional file 2: Table S2). Phylogenetic analysis of the NPFs from these different species highly supported that of *BnaNPFs* (Additional file 15: Figure S7). Moreover, the gene and protein structures (Additional file 4: Figure S2, Additional file 5: Figure S3, and Additional file 6: Figure S4) and expression patterns (Figs. 4 and 5) of *BnaNPFs* in each subfamily supported our classification, as well. Interestingly, all the NPFs involved in secondary metabolite transport known to date belong to the NPF2–1 subfamily (Table 1). We confirmed that 267 proteins belonging to this subfamily exist in 31 angiosperms, though not in the lower plants *P. patens* and *S. moellendorffii* [7] (Additional file 16: Table S9). Given that currently known members of the NPF2–1 subfamily across different plant species, including lower plants (Table 1), are mainly involved in nitrate transport, we speculated that the secondary metabolite transport feature of this subfamily was newly evolved in a given lineage or species in angiosperms during their evolution, indicating the specific subfunctionalization trend of this gene family.

## Conclusions

In this study, 199 *BnaNPFs* were identified in the *B. napus* genome and divided into 11 subfamilies having conserved gene and protein structures within each subfamily or clade. The allopolyploidy produced by its ancestors and the small-scale duplication events in *B. napus* acted as the primary driving forces for the massive expansion of this gene family in *B. napus*. Genes derived from *B. rapa* were retained after the allopolyploidy event during *B. napus* evolution. Most of the *BnaNPFs* were likely to be preferentially expressed in a few tissues or organs, and these expression profiles were commonly conserved in each subfamily or in each clade within a subfamily. Hormone inductions regulated the expression of many *BnaNPFs*. Five genes (*BnaNPF2.12*, *BnaNPF2.13*, *BnaNPF2.14, BnaNPF2.26,* and *BnaNPF2.28*) in the NPF2–1 subfamily may be involved in GLS transport in *B. napus*, mediated by IAA, ACC, GA_3_, or 6-BA.

## Methods

### Identification of NPF proteins in *B. napus* and phylogenetic analysis

The 53 AtNPFs were obtained from a previous report [20]. To identify NPFs in the *B. napus* genome, we performed a preliminary repeated BLASTP analysis against the proteome of *B. napus* (Darmor–*bzh* ecotype) in the GENOSCOPE database (http://www.genoscope.cns.fr/brassicanapus/) [27], using AtNPFs as queries (E-value < 1.0). Preliminary sequences were analyzed by Pfam (http://pfam.xfam.org/search/sequence) [28] and SMART (http://smart.embl-heidelberg.de/smart/show_motifs.pl) to ensure that the candidates had the typical PTR2 domain [29]. The DNA, CDS, and protein sequences of candidates were obtained from the GENOSCOPE database. Predictions of molecular weight and pI of candidates were performed using ProtParam (https://web.expasy.org/protparam/) [75]. To ensure reliability, the subcellular localization of BnaNPFs was predicted by Cell-PLoc2.0 (http://www.csbio.sjtu.edu.cn/bioinf/Cell-PLoc-2/) [31], PProwler version 1.2 (http://bioinf.scmb.uq.edu.au:8080/pprowler_webapp_1-2/index.jsp) [32], and WoLF PSORT (https://wolfpsort.hgc.jp/) [33] separately. Multiple sequence alignment of candidate protein sequences was performed using MAFFT version 7 online software with default parameters (https://mafft.cbrc.jp/alignment/server/) [34]. NJ trees were constructed with MEGA7.0 software [35] using a p-distance model and pairwise deletion, with a bootstrap analysis of 1000 replications. MEGA7.0 was also used to predict the best model for constructing the ML tree based on Bayesian information criterion (BIC) scores, then applied to construct the ML tree itself, using the bootstrap method (100 replications), JJT amino acid substitution with freqs. (+F) model, and gamma distribution shape parameter, based on the multiple sequence alignment. Trees were visualized and edited in Evolview V3 (https://www.evolgenius.info//evolview/#login). NPFs in the *B. oleracea* genome were identified in BRAD (http://brassicadb.org/brad/) by the same method [30]. NPF sequences in *O. sativa*, *P. trichocarpa*, *Z. mays*, *B. rapa,* and *G. max* were extracted from previous reports [7].

### Sequence feature analysis and regulatory gene prediction of NPFs in *B. napus*

Gene structures of candidate *BnaNPFs* and *AtNPFs* were investigated by GSDS 2.0 (http://gsds.gao-lab.org/) [38] with default parameters using the DNA sequence and CDS of candidates. The TMs and other protein domains of BnaNPFs and AtNPFs were predicted by HMMER V3.1b2 (http://www.ebi.ac.uk/Tools/hmmer) [36]. Potential *CREs* in upstream promoter regions (− 2000 bp) of candidate *BnaNPFs* were predicted by PlantCARE (http://bioinformatics.psb.ugent.be/webtools/plantcare/html/) [40]. The PlantTFDB database predicted TF binding sites in promoter sequences (− 2000 bp) of candidate BnaNPFs with default parameters (http://planttfdb.gao-lab.org/) [41].

### Chromosomal location and collinearity synteny analysis

The chromosome information of the 199 candidate *BnaNPFs* was obtained from the GENOSCOPE database. The collinear relationship of the candidate *NPF*s in *B. oleracea*, *B. rapa,* and *B. napus* genomes was assessed using the CoGe online software (https://genomevolution.org/CoGe/) [42] with default parameters. Duplication events of candidate *NPFs* were defined according to the method used in our previous report [76]. Based on cross-genome collinearity analysis, the species with the maximum orthologous blocks/most closer colinear relationships (including the NPF orthologous gene pairs) are considered the progenitors of *BnaNPFs*. HE, SE, and SD events were distinguished from each other based on chromosomal homology and colinear relationship (orthologous gene pairs in orthologous blocks) of the A_n_ (derived from *B. rapa*) and C_n_ (derived from *B. oleracea*) subgenomes and their respective progenitor genomes (*B. rapa* and *B. oleracea*) in all possible combination pairs. The chromosome map of candidate *BnaNPFs* was drawn by using the MapChart software with default parameters [77].

### Expression profile analysis of NPFs in Arabidopsis and *B. napus*

The expression profile of *AtNPFs* (including root, stem, leaf, apex, flower, and seed) was obtained from AtGenExpress (http://weigelworld.org/resources.html) [43]. The RNA-seq data, including 50 tissues of seven *B. napus* variety ZS11 organs (root, stem, leaf, flower, seed, and silique) at six developmental stages (seed germination, seedling, budding, initial flowering, full-bloom, and seed maturation), were obtained from NCBI (BioProject ID PRJNA358784). Expression profiles of candidate *BnaNPFs* in ZS11 seedling roots under five exogenous hormone treatments (IAA, GA_3_, 6-BA, ABA, and ACC) were extracted from our RNA-seq dataset (BioProject ID: PRJNA608211). *BnaNPFs* with FPKM ≥1 were retained, and the FPKM values of candidates were log2-transformed for visualization by the R package [78]. The heatmap was combined with hierarchical clustering methods of the log2-transformed RNA-Seq data [79].

### Plant material and hormone treatment

The seeds of a high-GLS content *B. napus* variety (Zhongyou 821; ZY821) and a low-GLS content variety (Zhongshuang 11; ZS11) were grown in Beibei (Chongqing, China) with standard agronomic procedures to analyze the temporal and spatial expression patterns of *BnaNPFs* involved in GLS transport. The root (Ro-s), stem (St-s), and leaf (Le-s) tissues at the seedling stage; the root (Ro-f), stem (St-f), leaf (Le-f), and flower (Fl-f) tissues at the flowering stage; and the root (Ro-ss), stem (St-ss), leaf (Le-ss), siliques 3 days after pollination (Si-3d), siliques 7 days after pollination (Si-7d), siliques 15 days after pollination (Si-15d), the seed 15 days after pollination (Se-15d), and seed 45 days after pollination (Se-45d) at the mature stage were collected from both ZS11 and ZY821. All tissues were immediately frozen in liquid nitrogen and stored at − 80 °C for RNA isolation.

To further analyze the expression patterns of the GLS transporter genes under five exogenous hormone inductions (ABA, IAA, GA_3_, 6-BA, and ACC), ZS11 seeds were germinated and cultivated at 25 °C under a 16/8 h (day/night) photoperiod in an artificial climate chamber. At the three-leaf stage, seedlings were transferred to Hoagland solution and were further cultured to the five-leaf stage. Seedlings were then treated with Hoagland’s liquid medium, containing 10 μmol/L IAA, 50 μmol/L ABA, 75 μmol/L 6-BA, 120 μmol/L GA_3_, and 60 μmol/L ACC. Root tissues were harvested at 0, 1, 3, 6, 12, and 24 h after each treatment. All samples were quickly frozen in liquid nitrogen and then stored at − 80 °C for RNA isolation.

### Expression analysis of NPFs in *B. napus* using qRT-PCR

The expression profile of 12 putative GLS transporters encoding *BnaNPFs* of the NPF2–1 subfamily in different tissues and under five exogenous hormone inductions was analyzed via qRT-PCR, using *BnaActin7* (GenBank accession no. AF024716) and *BnaUBI* (GenBank accession no. NC027770) as the reference genes. Primer pairs for qRT-PCR analysis were designed using Primer Premier 5 (Additional file 17: Table S10).

Total RNA was extracted from each of the samples with the EASYspin Total RNA Extraction Kit (Biomed, Beijing). The concentration and quality of total RNA for each sample were confirmed through gel electrophoresis analysis and NanoDrop 2000 spectrophotometer measurement. Potentially contaminating DNA was eliminated by DNase I (Promega, USA). The cDNA of each sample was synthesized using the M-MuLV RT kit (Takara Biotechnology, Japan). The real-time PCR analysis (qRT-PCR) was performed using the SYBR-Green PrimeScript RT-PCR Kit (Takara Biotechnology, Japan) with the CFX Connect™ Real-Time System (Bio-Rad, Chongqing, China). The parameters of qRT-PCR were as follows: 95 °C for 3 min (initial denaturation), followed by 40 cycles of 95 °C for 10s (denaturation) and 58 °C for 30s (annealing). Each PCR was validated in three independent repeat experiments. The qRT-PCR results were calculated using the 2^-∆∆ct^ method [47]. Expression values were log2-transformed and visualized with the R package [78].

## Supplementary Information

**Additional file 1: Table S1**. List of physical and chemical properties of candidate BnaNPF proteins.

**Additional file 2: Table S2**. Protein sequences and classification of NPF proteins in *Arabidopsis thaliana*, *Oryza sativa*, *Populus trichocarpa*, *Zea mays*, *Brassica rapa*, *Glycine max*, *Brassica oleracea*, and *Brassica napus* used in this study.

**Additional file 3: Figure S1**. Maximum likelihood (ML) tree of NPF proteins from *Brassica napus* and *Arabidopsis. (PDF 936 kb)*

**Additional file 4: Figure S2**. Protein structure of NPF proteins in *Brassica napus* and *Arabidopsis. (PDF 12899 kb)*

**Additional file 5: Figure S3**. The EXXEK(R) domain of NPF proteins in *Brassica napus* and *Arabidopsis. (PDF 4401 kb)*

**Additional file 6: Figure S4**. Gene structures of *NPF* genes in *Brassica napus* and *Arabidopsis. (PDF 4090 kb)*

**Additional file 7: Table S3**. List of *cis*-acting elements in promoter regions of *BnaNPF* genes.

**Additional file 8: Table S4**. Transcription factors with potential binding sites in promoter regions of *BnaNPF* genes.

**Additional file 9: Figure S5**. Chromosome distributions of *NPF* genes in *Arabidopsis*, *Brassica rapa,* and *Brassica oleracea. (PDF 1556 kb)*

**Additional file 10: Table S5**. Duplication events of *NPF* genes in *Brassica napus*, *Brassica rapa*, and *Brassica oleracea. (XLSX 23 kb)*

**Additional file 11: Table S6**. Expression profiles (FPKM value) of *BnaNPF* genes in 50 *Brassica napus* tissues at different developmental stages.

**Additional file 12: Figure S6**. Expression profiles of *Arabidopsis NPF* genes.

**Additional file 13: Table S7**. Expression profiles of 12 candidate *BnaNPF* genes in ZS11 and ZY821 by qRT-PCR.

**Additional file 14: Table S8**. Expression profiles of 12 candidate *BnaNPF* genes under hormone inductions by qRT-PCR.

**Additional file 15: Figure S7**. Neighbor-joining (NJ) tree of NPF proteins from *Arabidopsis*, *Oryza sativa*, *Populus trichocarpa*, *Zea mays*, *Brassica rapa*, *Brassica oleracea, Glycine max,* and *Brassica napus. (PDF 2332 kb)*

**Additional file 16: Table S9**. Distribution of the NPF2–1 subfamily members in 32 land plant species.

**Additional file 17: Table S10**. Primer list of the *BnaNPF* genes for qRT-PCR analysis.

## Data Availability

The data supporting the results of this study are included in manuscript and its additional files.

## Abbreviations

TM: Transmembrane domain
GLS: Glucosinolate
CREs: *Cis*-acting regulatory elements
TF: Transcription factor
SE: Segmental exchange
SD: Segmental duplication
HE: Homologous exchange
TD: Tandem duplication
ZS11: Zhongshuang 11
ZY821: Zhongyou 821
IAA: auxin
ABA: Abscisic acid
GA: Gibberellin acid
MeJA: Methyl jasmonate
6-BA: 6-benzylaminopurine
ACC: 1-aminocyclopropanecarboxylic acid
