# Supplementary material for: Genome-wide characterization, expression analyses, and functional prediction of the NPF family in Brassica napus
Source: BMC Genomics. 2020 Dec 7;21:871. doi: 10.1186/s12864-020-07274-7 (PMC7720588; doi:10.1186/s12864-020-07274-7)
Supplement: Supplementary file 3 — Additional file 3: Figure S1. Maximum likelihood (ML) tree of NPF proteins from Brassica napus and Arabidopsis. (PDF 936 kb) [file 12864_2020_7274_MOESM3_ESM.pdf]

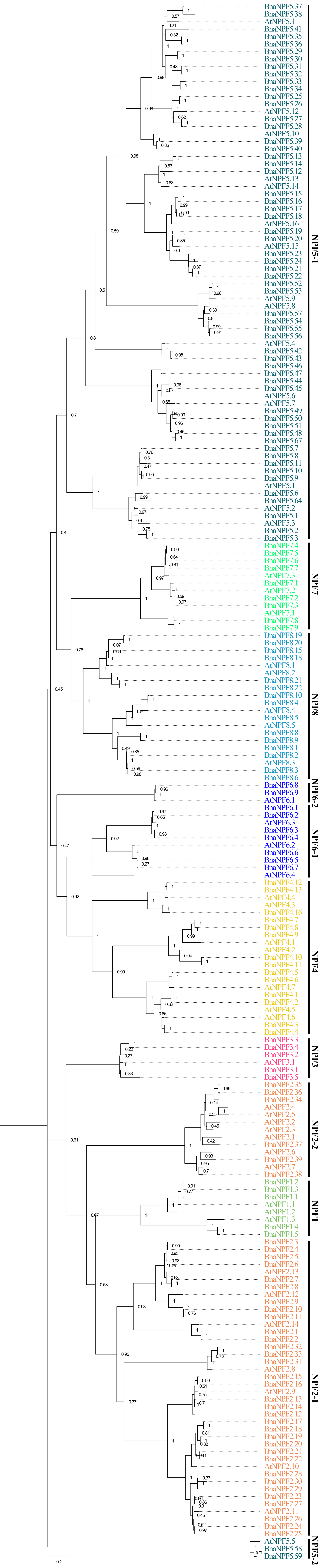

Additional file 3: Figure S1. Maximum likelihood (ML) tree of NPF proteins from *Brassica napus* and *Arabidopsis*. The phylogenetic tree was constructed using the 208 candidate NPF proteins from *Arabidopsis* (At: 53) and *B. napus* (Bna: 155). The NPF proteins were classified into 11 subfamilies based on the bootstrap value and topology of the ML tree. Bootstrap values are indicated as decimal along the branches in the ML tree.
