# Supplementary material for: Genome-wide characterization, expression analyses, and functional prediction of the NPF family in Brassica napus
Source: BMC Genomics. 2020 Dec 7;21:871. doi: 10.1186/s12864-020-07274-7 (PMC7720588; doi:10.1186/s12864-020-07274-7)
Supplement: Supplementary file 4 — Additional file 4: Figure S2. Protein structure of NPF proteins in Brassica napus and Arabidopsis. (PDF 12899 kb) [file 12864_2020_7274_MOESM4_ESM.pdf]

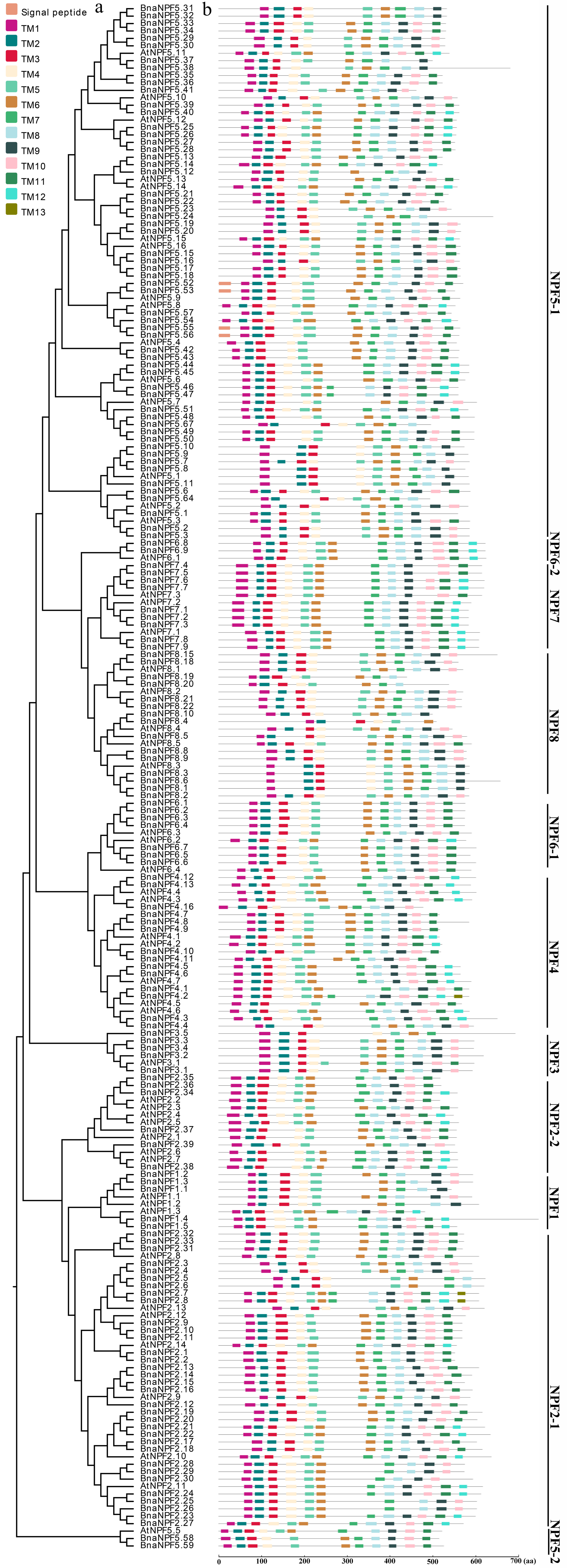

Additional file 4: Figure S2. Protein structure of NPF proteins in *Brassica napus* and *Arabidopsis*. (a)The NJ tree of *B. napus* and *Arabidopsis* NPF proteins. (b) Protein structures of *B. napus* and *Arabidopsis* *NPF* genes; Different colored boxs indicate the transmembrane regions (TMs) in the NPF proteins.
