# Supplementary material for: Genome-wide characterization, expression analyses, and functional prediction of the NPF family in Brassica napus
Source: BMC Genomics. 2020 Dec 7;21:871. doi: 10.1186/s12864-020-07274-7 (PMC7720588; doi:10.1186/s12864-020-07274-7)
Supplement: Supplementary file 5 — Additional file 5: Figure S3. The EXXEK(R) domain of NPF proteins in Brassica napus and Arabidopsis. (PDF 4401 kb) [file 12864_2020_7274_MOESM5_ESM.pdf]

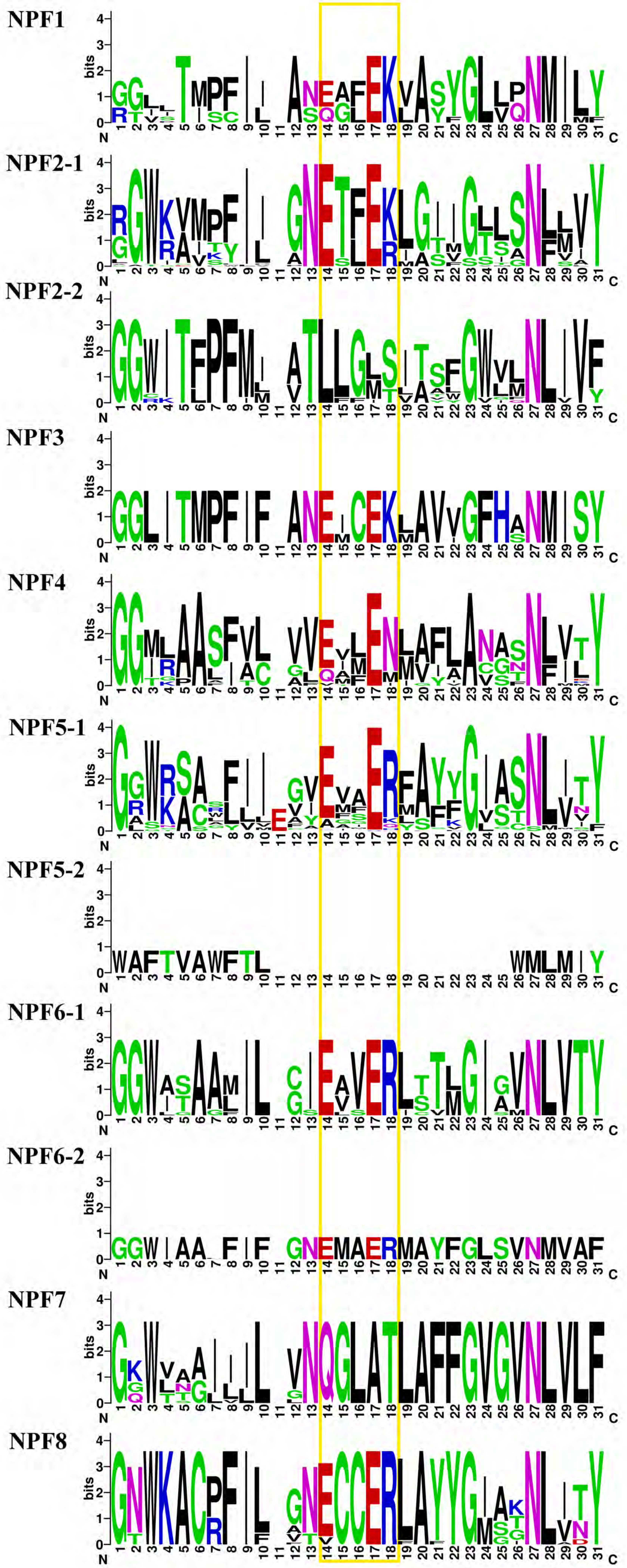

Additional file 5: Figure S3. **The EXXEK(R) domain of NPF proteins in *Brassica napus* and *Arabidopsis*.** The names of the 11 subfamilies of the NPF gene family are shown on the left. The yellow box indicates the EXXEK(R) domain.
