# Supplementary material for: Genome-wide characterization, expression analyses, and functional prediction of the NPF family in Brassica napus
Source: BMC Genomics. 2020 Dec 7;21:871. doi: 10.1186/s12864-020-07274-7 (PMC7720588; doi:10.1186/s12864-020-07274-7)
Supplement: Supplementary file 6 — Additional file 6: Figure S4. Gene structures of NPF genes in Brassica napus and Arabidopsis. (PDF 4090 kb) [file 12864_2020_7274_MOESM6_ESM.pdf]

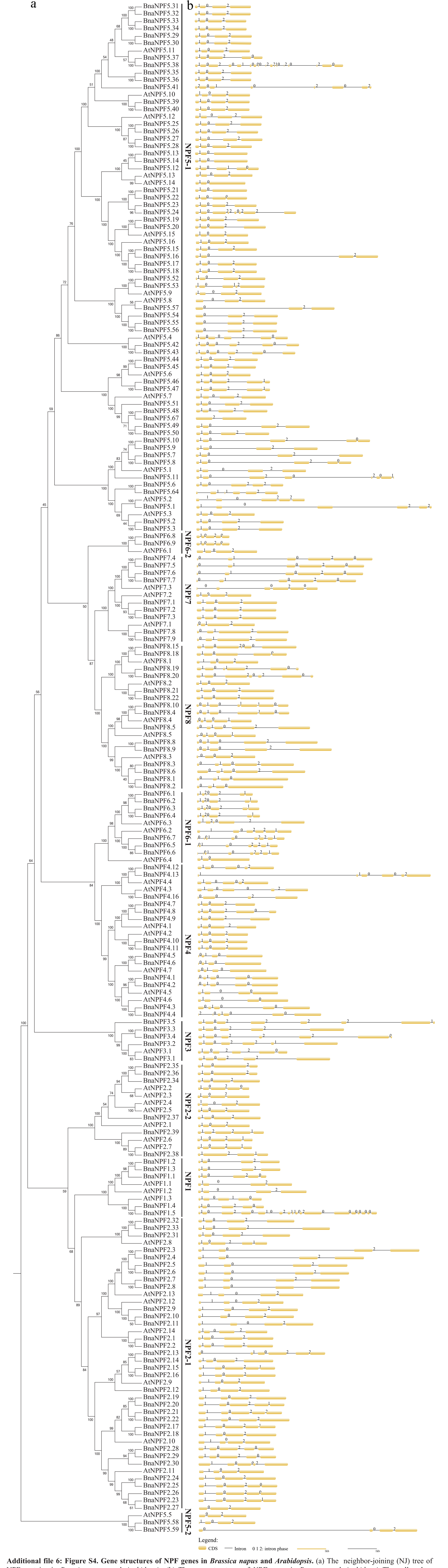

Additional file 6: Figure S4. Gene structures of NPF genes in *Brassica napus* and *Arabidopsis*. (a) The neighbor-joining (NJ) tree of NPF proteins in *Brassica napus* and *Arabidopsis*. (b) The gene structures of *NPF* genes in *B. napus* and *Arabidopsis*.The yellow box indicates the exon; the black lines connecting the exons represent the introns.
