# Supplementary material for: Genome-wide characterization, expression analyses, and functional prediction of the NPF family in Brassica napus
Source: BMC Genomics. 2020 Dec 7;21:871. doi: 10.1186/s12864-020-07274-7 (PMC7720588; doi:10.1186/s12864-020-07274-7)
Supplement: Supplementary file 9 — Additional file 9: Figure S5. Chromosome distributions of NPF genes in Arabidopsis, Brassica rapa, and Brassica oleracea. (PDF 1556 kb) [file 12864_2020_7274_MOESM9_ESM.pdf]

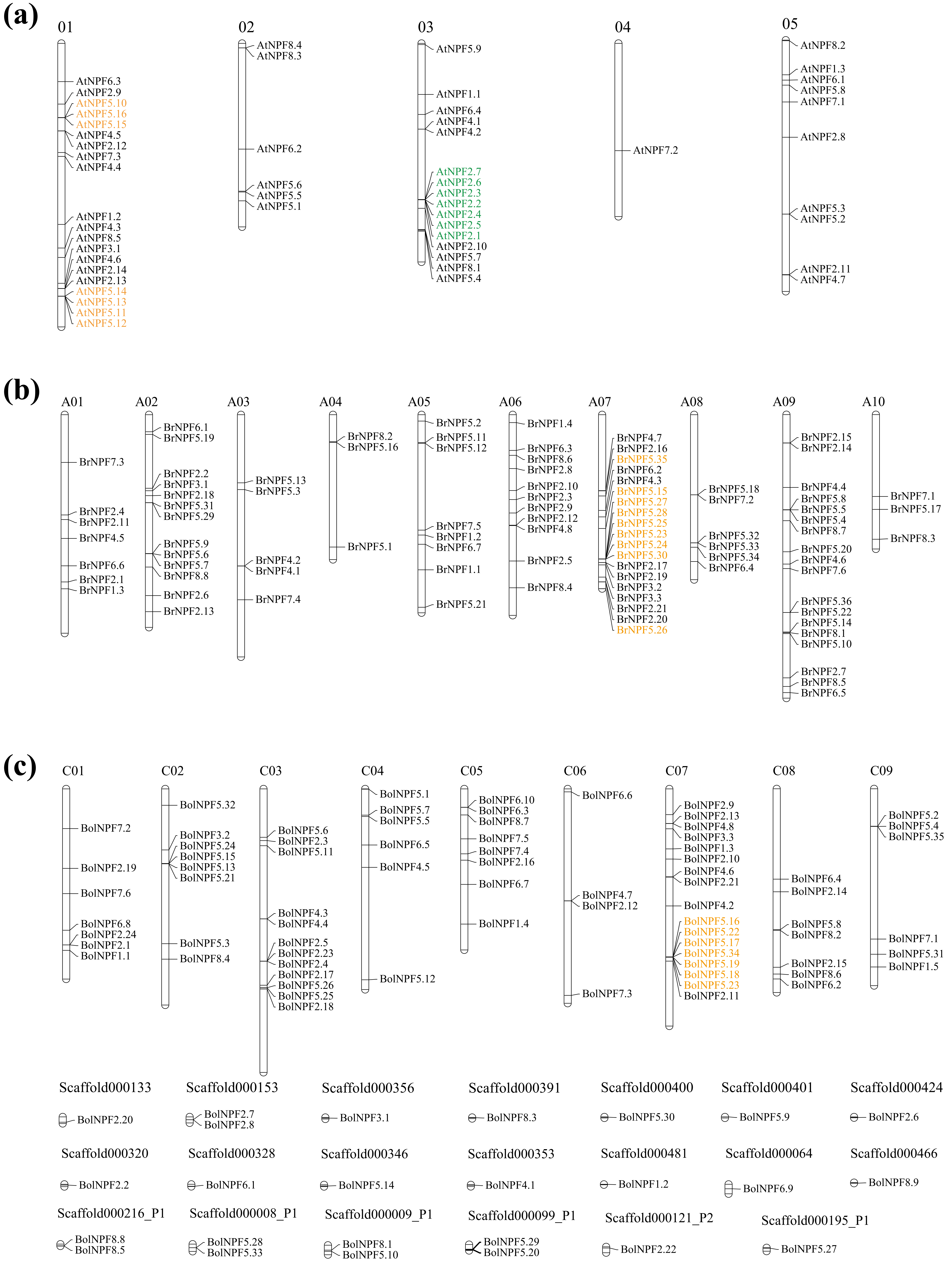

Additional file 9: Figure S5. Chromosome distributions of *NPF* genes in *Arabidopsis*, *Brassica rapa*, and *Brassica oleracea*. (a) The 53 *AtNPFs* were mapped on the five *Arabidopsis* chromosomes. **(b)** The 93 *BraNPFs* were mapped on the 10 *Brassica rapa* chromosomes. **(c)** The 100 *BolNPFs* were mapped on the nine *Brassica oleracea* chromosomes. Genes highlighted in orange indicate the members of the NPF5-1 subfamily that are clustered on a given chromosome. Genes highlighted in green indicate the members of the NPF2-2 subfamily that are clustered on the 03 chromosome in *Arabidopsis*.
