# Supplementary material for: Genome-wide characterization, expression analyses, and functional prediction of the NPF family in Brassica napus
Source: BMC Genomics. 2020 Dec 7;21:871. doi: 10.1186/s12864-020-07274-7 (PMC7720588; doi:10.1186/s12864-020-07274-7)
Supplement: Supplementary file 12 — Additional file 12: Figure S6. Expression profiles of Arabidopsis NPF genes. [file 12864_2020_7274_MOESM12_ESM.pdf]

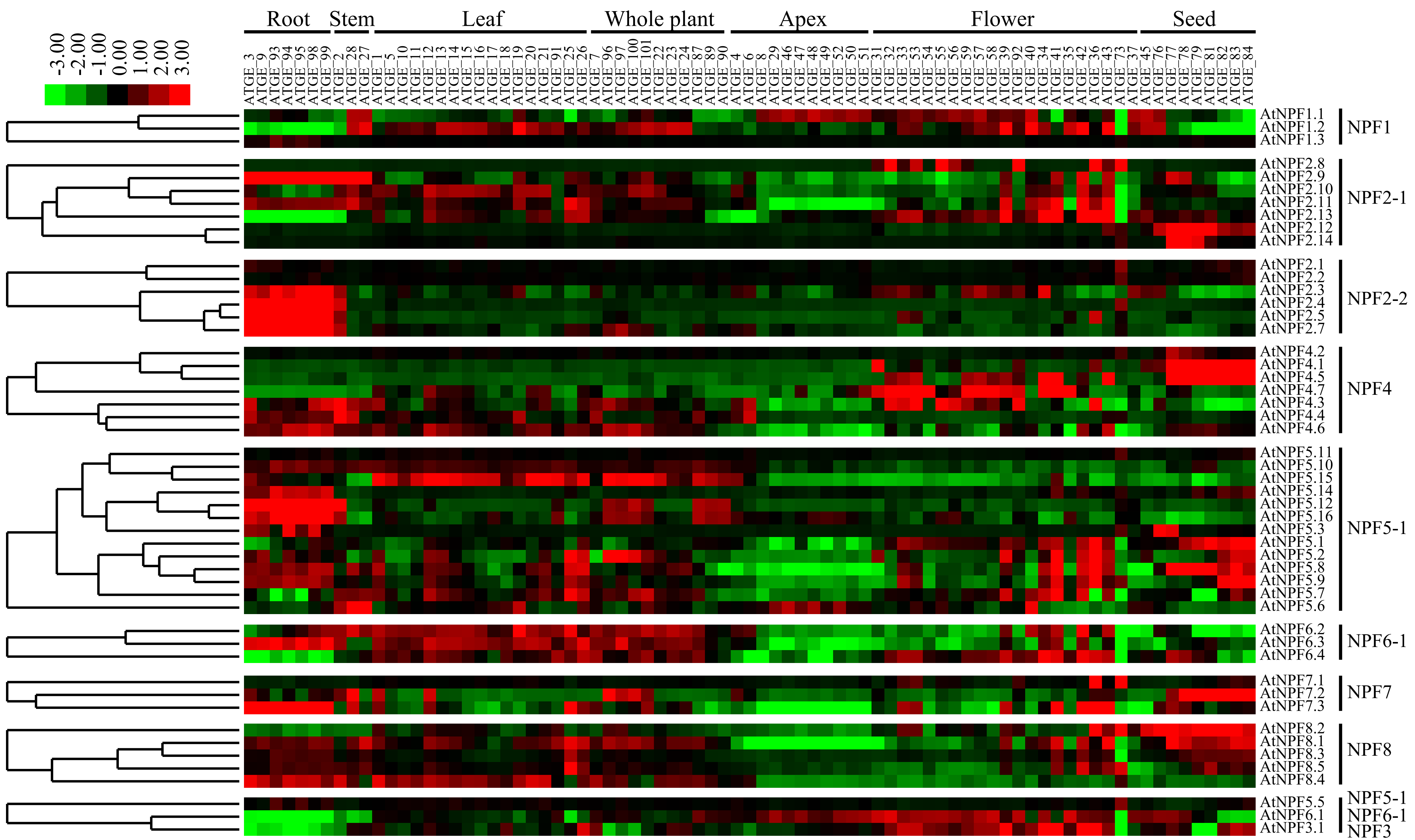


**Additional file 12: Figure S6. Expression profiles of *Arabidopsis NPF* genes.** The expression profiles of the 50 *AtNPFs* in six organs (root, stem, leaf, apex, flower and seed). The colour bar at the top represents log2 expression value.
