# Supplementary material for: Genome-wide characterization, expression analyses, and functional prediction of the NPF family in Brassica napus
Source: BMC Genomics. 2020 Dec 7;21:871. doi: 10.1186/s12864-020-07274-7 (PMC7720588; doi:10.1186/s12864-020-07274-7)
Supplement: Supplementary file 15 — Additional file 15: Figure S7. Neighbor-joining (NJ) tree of NPF proteins from Arabidopsis, Oryza sativa, Populus trichocarpa, Zea mays, Brassica rapa, Brassica oleracea, Glycine max, and Brassica napus. (PDF 2332 kb) [file 12864_2020_7274_MOESM15_ESM.pdf]

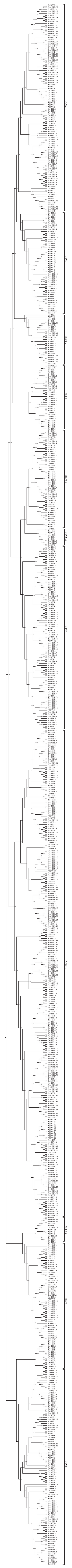

Additional file 15: Neighbor-joining (NJ) tree of NPF proteins from *Arabidopsis*, *Oryza sativa*, *Populus trichocarpa*, *Zea mays*, *Brassica rapa*, *Brassica oleracea*, *Glycine max*, and *Brassica napus*. A total of 836 NPF proteins are used in the NJ tree, including At: 53, Os: 93, Pt: 85, Zm: 79, Br: 94, Bo: 100, Gm: 133 and Bn: 199. The candidate NPF proteins were divided into 11 subfamilies.
